# Influence of Light Conditions on the Antibacterial Performance and Mechanism of Waterborne Fluorescent Coatings Based on Waterproof Long Afterglow Phosphors/PDMS Composites

**DOI:** 10.3390/polym15193873

**Published:** 2023-09-24

**Authors:** Sinan Hao, Yuhong Qi, Zhanping Zhang

**Affiliations:** 1 Key Laboratory of Ship-Machinery Maintenance & Manufacture, Dalian Maritime University, Dalian 116026, China; hsn@dlmu.edu.cn (S.H.); zzp@dlmu.edu.cn (Z.Z.); 2 Department of Materials Science and Engineering, Dalian Maritime University, Dalian 116026, China

**Keywords:** polydimethylsiloxane, long afterglow phosphor, waterborne, fluorescence, fouling-release, marine bacteria

## Abstract

Marine microbial adhesion is the fundamental cause of large-scale biological fouling. Low surface energy coatings can prevent marine installations from biofouling; nevertheless, their static antifouling abilities are limited in the absence of shear forces produced by seawater. Novel waterborne antifouling coatings inspired by fluorescent coral were reported in this paper. Waterproof long afterglow phosphors (WLAP) were introduced into waterborne silicone elastomers by the physical blending method. The composite coatings store energy during the day, and the various colors of light emitted at night affect the regular physiological activities of marine bacteria. Due to the synergistic effect of fouling-release and fluorescence antifouling, the WLAP/polydimethylsiloxane (PDMS) composite coating showed excellent antifouling abilities. The antibacterial performance of coatings was tested under simulated day-night alternation, continuous light, and constant dark conditions, respectively. The results illustrated that the antibacterial performance of composite coatings under simulated day-night alternation conditions was significantly better than that under continuous light or darkness. The weak lights emitted by the coating can effectively inhibit the adhesion of bacteria. C-SB/PDMS showed the best antibacterial effect, with a bacterial adhesion rate (BAR) of only 3.7%. Constant strong light also affects the normal physiological behavior of bacteria, and the weak light of coatings was covered. The antibacterial ability of coatings primarily relied on their surface properties under continuous dark conditions. The fluorescent effect played a vital role in the synergetic antifouling mechanism. This study enhanced the static antifouling abilities of coatings and provided a new direction for environmentally friendly and long-acting marine antifouling coatings.

## 1. Introduction

Microbial adhesion and large-scale biological pollution on the surface of ships and marine facilities have become a global problem in the marine industry, causing serious losses to the marine economy [1,2,3]. Biological fouling not only increases the mass and surface roughness of the hull, leading to increased friction and energy consumption during navigation, but even reduces the accuracy of marine monitoring equipment, resulting in the distortion of them [4,5,6,7]. The growth of fouling organisms can accumulate up to 150 kg/m^2^ on unprotected hulls in the ocean in half a year [8]. At present, antifouling coatings are considered to be the most effective and economical strategy among various antifouling technologies, providing long-term protection for ships and offshore engineering equipment in harsh marine environments [9].

Marine biofouling can generally be divided into two main stages, namely the formation of micro-fouling and macro-fouling [7]. In the microscopic fouling stage, various bacteria and microorganisms adhere to the surface of marine facilities while secreting extracellular polymeric substances (EPS) to form microbial membranes. During the large pollution phase, the microbial membrane induces the adhesion of algae and protozoa and then further attracts the attachment and colonization of larva of large fouling organisms. Therefore, inhibiting the initial adhesion of bacteria is the key to preventing biofouling. Marine bacteria, as prokaryotic single-celled organisms that do not contain chlorophyll and phycocyanin, are the most widely distributed and abundant marine microorganisms [10]. There are various types such as autotrophic and heterotrophic, phototrophic and chemotrophic, parasitic and saprophytic, aerobic and anaerobic, and planktonic and attached. Gram-negative bacteria predominate in seawater, while Gram-positive bacteria predominate in ocean bottom sediments. In general, 10^2^–10^3^ bacterial colonies can be isolated from each milliliter of coastal seawater, sometimes more than 10^5^. Some marine bacteria have the ability to photosynthesize [11].

As an ideal marine antifouling material, polydimethylsiloxane (PDMS)-based coating has the properties of a smooth surface, low elastic modulus, and low surface free energy (SFE), which contributes to its fouling-release performance [12,13]. However, the fouling-release process depends extremely on the scour of water, and its static antifouling abilities are very limited. In recent years, bioinspired marine antifouling coatings have become an international research hotspot with the development of non-toxic and eco-friendly antifouling materials [1,14]. Natural antifouling agents and their synthetic analogs from various organisms [15] and the strategy of a slippery liquid-infused porous surface (SLIPS) have been widely studied [16,17]. However, the large-scale commercial application of natural antifouling agents is challenging since the extraction process is costly and complex and may upset the ecological balance, and the service life of SLIPS is very limited due to the poor controllability of surface lubricants, which can lead to decreased antifouling performance.

By contrast, fluorescent corals, which have active surfaces and unique fluorescence effects, have attracted the attention of researchers [18,19,20]. They contain fluorescent proteins that protect themselves and emit faint light in a variety of colors, including red, yellow, green, blue, etc. in the deep-sea environment [21]. These weak lights can provide energy for the photosynthesis of photosynthetic microorganisms and effectively inhibit their adhesion, thus achieving antifouling effects [22]. Therefore, the introduction of fluorescent materials into antifouling coatings can effectively inhibit the initial adhesion of microorganisms. However, for the purpose of applying them in a complex marine environment, long-term performance and durability are important criteria. Related studies have introduced coumarin as an antifouling group into PDMS-based polyurethane coatings, which has the synergetic antifouling mechanism of contact antibacterial effects and fluorescence [23]. However, the process of extracting coumarin from natural organisms is complicated and costly. Several photocatalysts, such as g-C_3_N_4_ and TiO_2_, have photo-response effects, and the antifouling effect can be achieved through the reactive oxygen species (ROS) sterilization mechanism [24,25,26]. However, this photocatalytic effect can only be carried out during the day and lacks strong antifouling abilities at night when fouling organisms are more likely to adhere.

Hence, the selection of suitable fluorescent materials is very necessary. In recent years, as a kind of energy storage material, long afterglow phosphors have shown unique charm in the field of solar energy conversion and utilization, as they can store solar energy during the day and release it slowly at night, and the afterglow time can reach more than 10 h [27]. Meanwhile, the ultraviolet band of sunlight has a strong transmission in seawater [28]. Therefore, the application of this material in antifouling coatings can make the coating emit weak light at night, affect the normal physiological activities of photosynthetic microorganisms, and inhibit their adhesion. Inspired by the antifouling strategies of Gulf parrotfish and fluorescent corals, Jin et al. [29] introduced different colored phosphors into the PDMS matrix to prepare novel sandwich-structure coatings. The top silicone rubber layer was treated by ultraviolet ozone to obtain hydrophilicity. The coatings can effectively inhibit the initial adhesion of bacteria and algae under the synergistic effect of hydrophilicity and fluorescence. Nevertheless, the top layer may block the fluorescence of the middle layer, which reduces the luminous intensity of the composite coatings. Cao et al. [22] prepared a series of composite coatings of silicone elastomer and phosphors of various colors and investigated the adhesion of diatoms on their surface. The results showed that the composite coatings could not only inhibit the initial adhesion of diatoms but also maintain the fouling-release performance. However, these studies focused on solvent-based coatings, which produce volatile organic compounds (VOCs) with certain toxicity during the process of production and application and cause damage to the marine environment [30,31]. With the increasing awareness of marine ecology around the world, waterborne coatings have become a current development trend and established a new approach to developing eco-friendly antifouling coatings.

In this work, a series of waterborne fluorescent coatings were prepared using waterproof long afterglow phosphors (WLAP) with different wavelengths and luminous intensity and hydroxy-terminated silicone oil emulsion (HT-SOE), which have strong synergistic effects of fouling-release and fluorescence antifouling. The antibacterial mechanism of WLAP/PDMS coatings was investigated under different light conditions. This study opens up a new approach to the development of eco-friendly, long-acting, and broad-spectrum marine antifouling coatings.

## 2. Materials and Methods

### 2.1. Materials

Guangzhou Huicai Printing Pigment Co., Ltd. (Guangzhou, China) produced WLAP (purity of 99.9%) with a variety of luminous colors, including sky-blue, blue-green, orange-red, yellow-green, and lemon-yellow. They were marked P-SB, P-BG, P-OR, P-YG, and P-LY, respectively, for the purpose of discussion and analysis, where the first letter P stands for powders. A cationic HT-SOE (JF-5096), which was supplied from Jiashan Jiangnan Textile Material Co., Ltd. (Jiaxing, China), was the film-forming substance. The fillers and pigments were titanium dioxide (Anatase, TLA-100), hydrophilic fumed silica (AEROSIL A200), and talc (CMS444), which were produced by Sichuan Panzhihua Tianlun Chemical Co., Ltd. (Panzhihua, China), Evonik Special Chemical (Shanghai) Co., Ltd. (Shanghai, China), and Heshan Chemical (Liaoning) Co., Ltd. (Anshan, China), respectively. Tetraethyl orthosilicate (TEOS) and dibutyltin dilaurate (DBTDL) were purchased from Tianjin Damao Chemical Reagent Factory (Tianjin, China).

### 2.2. Preparation of WLAP/PDMS Composite Coatings

The following steps are taken to prepare the three-component WLAP/PDMS composite coatings. The powders used in the preparation were dried in an electric thermostatic drying oven (Jinghong DHG-9023A, Shanghai, China) at 80 °C for 48 h before use. In the first phase, the system was mechanically dispersed for 5 min at 500 rpm using deionized water (100 g) and additives (10 g) by a multi-function mixing-sanding-dispersing machine (Biuged BGD750, Guangzhou, China). Next, talc powder (40 g), fumed silica (3 g), and titanium dioxide powder (30 g) were stepwise added and mechanically dispersed for 30 min at 1000 rpm to create a uniform slurry. A tenth of the slurry was then added to a metal mixing tank containing 60 g HT-SOE, and the system was mechanically dispersed for 10 min at 600 rpm. Finally, WLAP (20 g) was added and mechanically dispersed for 10 min at 600 rpm to achieve the WLAP/PDMS composite paint. The paint served as component A, TEOS served as component B, and DBTDL served as component C. In the second phase, components A and TEOS were mixed equally, and DBTDL was then added and manually stirred for 5 min. The paint was obtained after the mixture was finished. The mixing mass ratio of HT-SOE in component A, TEOS, and DBTDL was (100:4:0.6). The preparation of coatings was the last phase. The paint was coated on glass slides measuring 25.4 mm × 76.2 mm × 1 mm, poured into a polytetrafluoroethylene (PTFE) mold measuring 100 mm × 90 mm × 5 mm, and then cured for 24 h at room temperature. The WLAP/PDMS composite coatings were prepared after curing. For convenience, the coatings were designated as C-xy/PDMS, where C stands for coating, and xy represents the color of WLAP, including SB, BG, OR, YG, and LY. The coating without WLAP served as the blank group and was named C-B/PDMS.

### 2.3. Characterization

#### 2.3.1. Crystal Structure

An X-ray diffractometer (XRD, Rigaku Smartlab, Nagano, Japan) was used to examine the crystal structure of WLAP. To qualitatively assess the crystal structure of WLAP, their measured diffraction spectra were compared with PDF reference cards using MDI Jade 6.5 software. The test settings were 10–70° test range, 10°/min scanning speed, and 0.01° steps. The experiments were run at room temperature on a copper target.

#### 2.3.2. Surface Morphology, Chemical Composition and Roughness

A field emission scanning electron microscope (SEM, ZEISS SUPRA 55 SAPPHIRE, Oberkochen, Germany) operating at 3 kV was utilized to observe the surface morphology of WLAP, and an energy dispersive spectrometer (EDS) operating at 20 kV was used to examine the elemental components of WLAP. The surface morphology of coatings was tested by an SEM (JEOL JSM-7610F Plus, Tokyo, Japan) operating at 5 kV. Using a confocal laser scanning microscope (CLSM, OLYMPUS OLS4000, Tokyo, Japan), the coating’s surface morphology was gathered and examined, and its surface roughness (R_a_) was determined using the LEXT program. Three samples were used to assess each coating, and the R_a_ of each sample was estimated separately. The average of test results was selected.

#### 2.3.3. Laser Particle Size Analysis

The specific surface area and median particle size (D50) of WLAP were examined using a laser particle size analyzer (Baite BT-9300SE, Dandong, China). Deionized water was used as the dispersion medium in a wet test. Before testing, the samples were spread out using an ultrasonic machine for 10 min. Each sample was examined three times, and the average was used to determine the outcome of the experiment.

#### 2.3.4. Luminescence Test

The fluorescence emission spectra of WLAP/PDMS composite coatings and WLAP with a 370 nm excitation wavelength were measured using a steady state/transient photoluminescence spectrometer (Edinburgh FLS1000, Scotland, England). The tested wavelength range was 400–650 nm. The afterglow illuminance of coatings was measured using a weak light illuminometer (Aobodi ST-900, Beijing, China), along with changes in its regularity over time. Before testing, the coatings were stimulated for 30 min under 3000 lx of illumination.

#### 2.3.5. Contact Angle and SFE

A contact angle meter (Zhongchen JC2000C, Shanghai, China) was used to measure coatings’ contact angles using the pendant-drop technique. The coating was dropped with 3 μL of diiodomethane and 3 μL of deionized water, respectively, and a snapshot was taken after 3 s. Before the test, the samples were ultrasonically cleaned with absolute ethyl alcohol for 5 min and with deionized water for 5 min to make sure the surface of the samples was clean and then dried for contact angle measurement to remove the influence of small molecular substances still on the coating surface, such as the emulsifier. The contact angle between two liquids with different polarities was calculated as the average of five parallel tests. Ultimately, the SFE was resolved using Owens’s two-liquid approach [32], as shown in Formulas (1) and (2),

(1) $$\gamma_{L}(1+\cos\theta )=2(\gamma_{s}^{d}{\text{…}\gamma_{L}^{d})}^{1/2}+2(\gamma_{s}^{p}{\text{…}\gamma_{L}^{p})}^{1/2}$$

(2) $$\gamma_{s}=\gamma_{s}^{d}+\gamma_{s}^{p}$$

where θ is the contact angle between the liquids and coatings. $\gamma_{L}$, $\gamma_{L}^{d}$, and $\gamma_{L}^{p}$ are the surface tension, dispersion force, and polarity force of the liquids, respectively. $\gamma_{s}^{d}$ and $\gamma_{s}^{p}$ are dispersion force and polarity force of the solid, that is, the coatings, respectively. $\gamma_{s}$ is the SFE of coatings.

By substituting the $\gamma_{L}$, $\gamma_{L}^{d}$, $\gamma_{L}^{p}$, and θ values of water and diiodomethane into formula (1) and solving the equations simultaneously, the $\gamma_{s}^{d}$ and $\gamma_{s}^{p}$ in the SFE of coatings can be obtained. The SFE ($\gamma_{s}$) of coatings was then obtained according to Formula (2). The $\gamma_{L}$, $\gamma_{L}^{d}$, and $\gamma_{L}^{p}$ of water and diiodomethane are given in Table S1.

#### 2.3.6. Tensile Test

An electronic universal testing machine with microprocessor control (Wance UTM5105, Jinan, China) was used to examine the coating’s mechanical properties. A dumbbell-shaped tensile sample of 75 mm by 4 mm was cut out of the coating in the polytetrafluoroethylene mold. The tensile speed was 50 mm/min. The gauge’s initial length was 25 mm. Finally, the coatings’ stress-strain curve, fracture elongation, and breaking strength were determined. The portion of the stress-strain curve that was closest to a straight line was chosen for the linear fitting, and the slope of the line can roughly be used to determine Young’s modulus of coatings.

#### 2.3.7. Evaluation of Antibacterial Effect

The ability of the coating to resist the adhesion of marine microorganisms was evaluated by the bacterial adhesion test. The experimental process was as follows. First, fresh seawater (Yellow Sea area, Dalian, China) was diluted 10^−3^ times. Nine slide samples of each coating were taken, and each slide sample was completely immersed in a Petri dish containing 40 mL diluted seawater for 24 h. The incubation conditions in the soaking process were divided into three groups: alternative incubation in 12 h light (3000 lx) and 12 h dark, 24 h constant light (3000 lx) environment, and 24 h continuous dark condition. Three slide samples of each coating were taken under each incubation condition. Each slide sample was taken out of the Petri dish 24 h later, thoroughly rinsed with 30 mL sterile seawater, placed into a centrifuge tube containing 40 mL sterile seawater, and then cleaned with an ultrasonic cleaner for 5 min. Next, the bacterial solution in the centrifuge tube was shaken well, and 100 μL bacterial solution was taken and equally distributed over the 2216E solid medium. Ultimately, the culture mediums were incubated for 24 h at 25 °C in a biochemical incubator, and the colony counts on the mediums were noted. The instruments used in the aforementioned experiments were disinfected under ultraviolet lamp irradiation or high temperature and pressure conditions before the experiment.

The calculation formula of bacterial adhesion rate (BAR) is shown in Formula (3),

BAR = R_c_/R_s_ × 100% (3)

where R_c_ and R_s_ are the number of bacteria on the WLAP/PDMS composite coatings and control group (slides), respectively.

## 3. Results and Discussion

### 3.1. Crystal Phase Composition of WLAP

**Figure 1:**
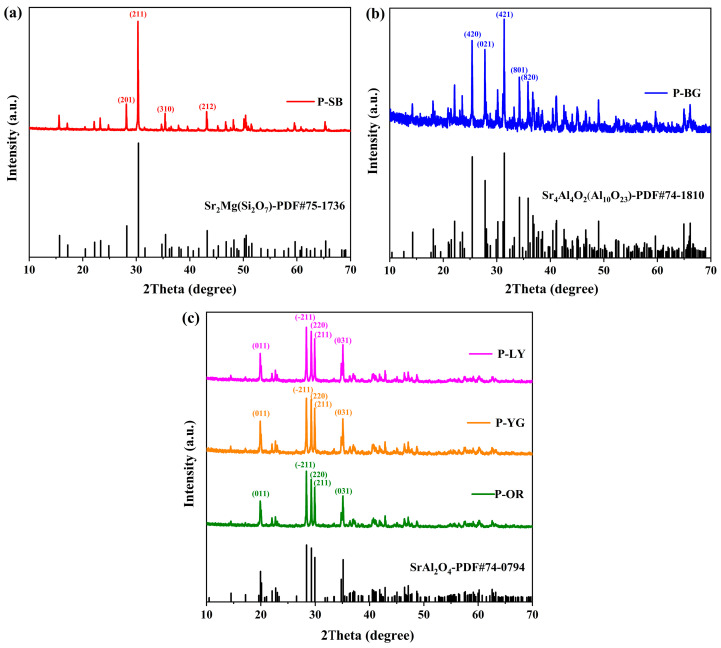
XRD patterns of (**a**) P-SB, (**b**) P-BG, and (**c**) P-OR, P-YG, and P-LY.

It is possible to determine the crystal structure of WLAP by qualitatively examining their phase composition. Figure 1 presents the XRD patterns of WLAP. The diffraction peaks of WLAP are in good agreement with the PDF standard cards. By comparing with the PDF standard card, it can be concluded from Figure 1a that the crystal substance of P-SB is Sr_2_Mg(Si_2_O_7_), which is a rare earth silicate phosphor. The diffraction peaks are in good agreement with the standard card of Sr_2_Mg(Si_2_O_7_), and the diffraction peaks appearing at the diffraction angles of 28.2°, 30.3°, 35.4°, and 43.2° correspond to the crystal planes of (201), (211), (310), and (212), respectively. Similarly, Figure 1b shows that the crystal material of P-BG is Sr_4_Al_4_O_2_(Al_10_O_23_), which belongs to a rare earth aluminate phosphor (REAP). The crystal structures of P-OR, P-YG, and P-LY are SrAl_2_O_4_, which also belongs to a type of REAP, as can be concluded from Figure 1c. Their diffraction peaks at 19.9°, 28.4°, 29.3°, 29.9°, and 35.1° correspond to the (011), (−211), (220), (211), and (031) crystal planes of the PDF standard card of SrAl_2_O_4_, respectively.

### 3.2. Surface Morphology and Particle Size

**Figure 2:**
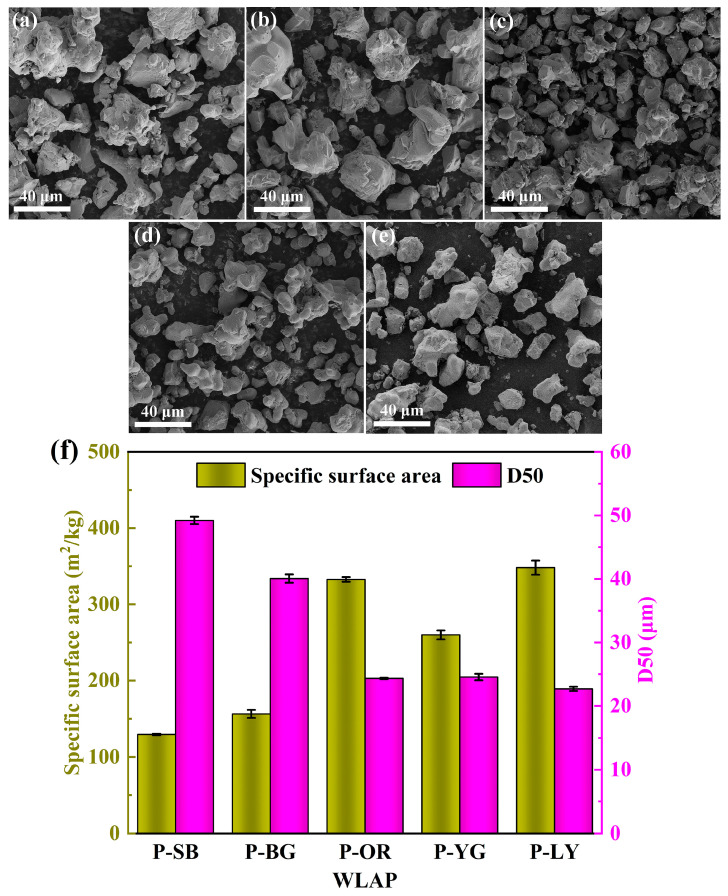
SEM image of (**a**) P-SB, (**b**) P-BG, (**c**) P-OR, (**d**) P-YG, and (**e**) P-LY; (**f**) Laser particle size analysis of WLAP.

The morphologies of WLAP were evaluated by SEM for the purpose of visual inspection of the surface morphology, and the images are given in Figure 2a–e. It can be observed that all WLAP were micron powders that were 25–50 μm in size. The five kinds of WLAP were distributed unevenly and had varying sizes and forms. Figure 2c–e are the images of powders with the same crystal structure (SrAl_2_O_4_), which had similar particle sizes and distribution laws. In order to determine the particle size of WLAP more accurately and explore the influence of particle size on the performance of coatings, the median particle size (D50) and specific surface area (SSA) of WLAP were examined by a laser particle size analyzer, as demonstrated in Figure 2f. It can be observed that P-SB had the largest D50, which was 49.2 μm. Moreover, there was a certain relevance between D50 and the SSA; that is, the SSA decreases when D50 increases. The size distribution curves of WLAP and HT-SOE are further presented in Figure S1, and the average particle sizes are given in Table S2. It can be seen that the D50 was close to the volumetric average particle size. The elemental composition of WLAP was examined by an EDS operating at 20 kV, as shown in Figure S2. It can be seen that the surface of P-SB contained O, Si, Mg, and Sr elements, and the surface of P-BG, P-OR, P-YG, and P-LY contained O, Al, and Sr elements.

### 3.3. Surface Performance of Coatings

**Figure 3:**
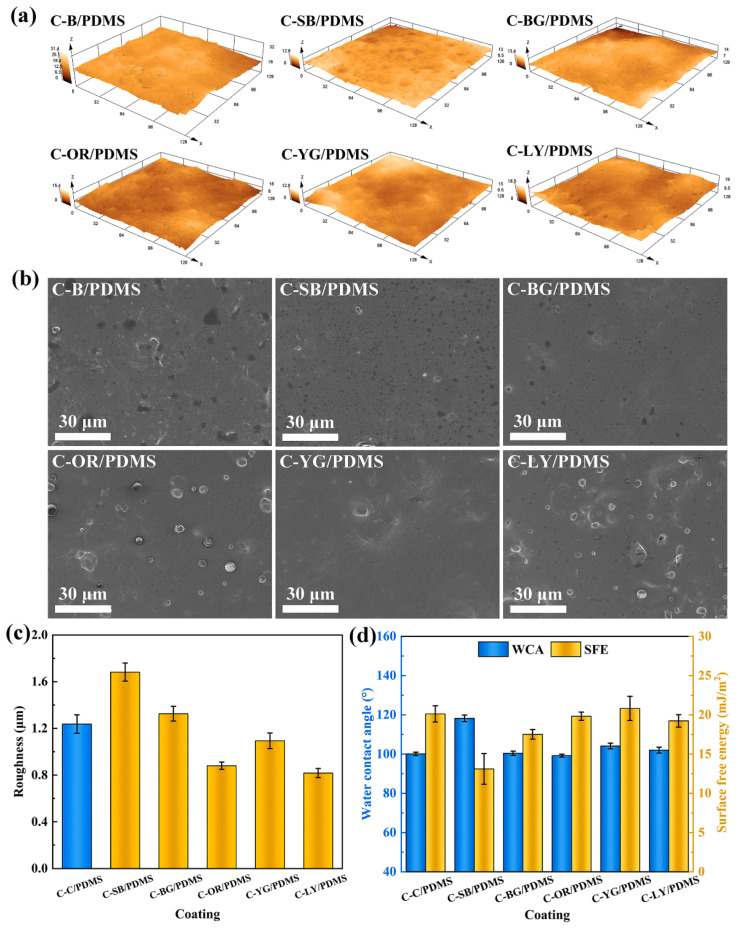
(**a**) CLSM images, (**b**) SEM images, (**c**) surface roughness (R_a_), and (**d**) water contact angle (WCA) and surface free energy (SFE) of coatings.

To explore the impacts of WLAP on the surface performance of coatings, the surface morphology of the coatings was determined by CLSM and SEM, and the wettability and SFE of the coatings were examined using a contact angle measuring instrument (JC2000C), as presented in Figure 3. It can be observed from the three-dimensional images of coatings (Figure 3a) that the introduction of WLAP affected the surface morphology of the coatings to different degrees. An SEM was used to further confirm the surface morphology of the coatings. It can be seen from Figure 3b that the micron particles were uniformly dispersed on the matrix of coatings by physical blending. In addition, there were some micropores on the surface of the coatings. This was due to the air microbubbles formed in the mechanical mixing process of the coating escaping during curing, resulting in the existence of micropores on the coatings. The results of the roughness (R_a_) calculations are given in Figure 3c. The variation in particle size of WLAP contributes to the distinction in R_a_. It can be noticed that the R_a_ of C-SB/PDMS and C-BG/PDMS was higher than C-B/PDMS, while that of other samples was lower. This is due to the particle size of the filler talc (400 mesh approximately 37 μm) being lower than that of P-SB and P-BG while being higher than that of other powders. The R_a_ of coatings somewhat changed after substituting WLAP for a particular quality of filler. It is generally recognized that the chemical composition and surface roughness of coatings have an impact on the wettability of coatings. Hence, the water contact angle (WCA) results (Figure 3d) demonstrated that the high roughness resulted in the high WCA, which fits the Cassie model [33]. This is owing to the rough surface retaining more air between the surface and the water droplet, thus promoting hydrophobicity [34]. The diiodomethane contact angles (DCAs) are given in Table S3. The SFE of the coatings, which was calculated by the contact angle, is given in Figure 3c. It can be seen that the SFE of the coatings was between 13.1 mJ/m^2^ and 20.8 mJ/m^2^. For PDMS-based coatings, a low SFE is beneficial to prevent the initial adhesion of fouling organisms or make them adhere weakly so that they are easy to remove under the shear of water.

### 3.4. Mechanical Properties of Coatings

**Figure 4:**
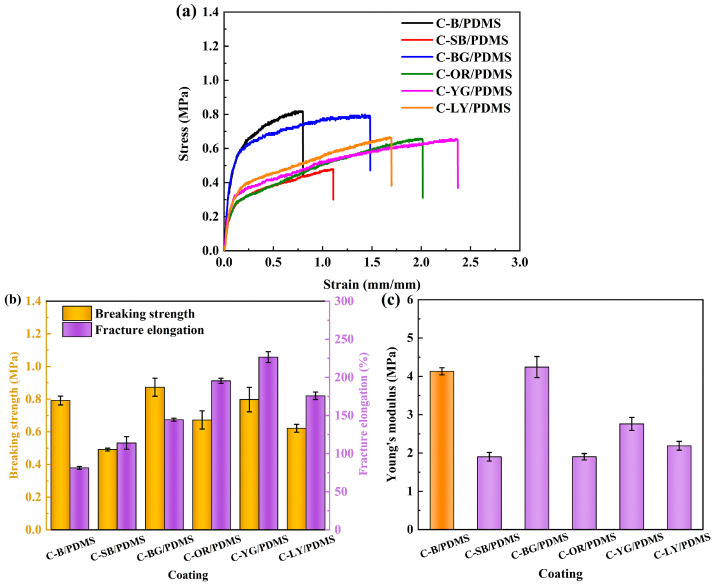
(**a**) Stress-strain curve, (**b**) breaking strength and fracture elongation, and (**c**) Young’s modulus of coatings.

The mechanical properties of the coating were analyzed, and the stress-strain curve is given in Figure 4a. Combined with Figure 4b, it can be seen that the introduction of WLAP enhanced the fracture elongation of the coatings and improved their flexibility. The fracture elongation of C-YG/PDMS was the highest, reaching 226.5%, which was more than three times higher than that of C-B/PDMS. However, the tensile strength of the composite coatings decreased to a value between 0.49 MPa and 0.8 MPa. It can be concluded from Figure 4c that the WLAP /PDMS composite coatings had a lower Young’s modulus than C-B/PDMS, less than 4.2 MPa. The lower modulus was conducive to removing fouling organisms from the surface of coatings, according to previous research [35,36]. The attached fouling organisms on the coatings with a low elastic modulus could easily be removed by stripping, while those on the coatings with a high elastic modulus were more inclined to shear off.

### 3.5. Luminescence Performance

**Figure 5:**
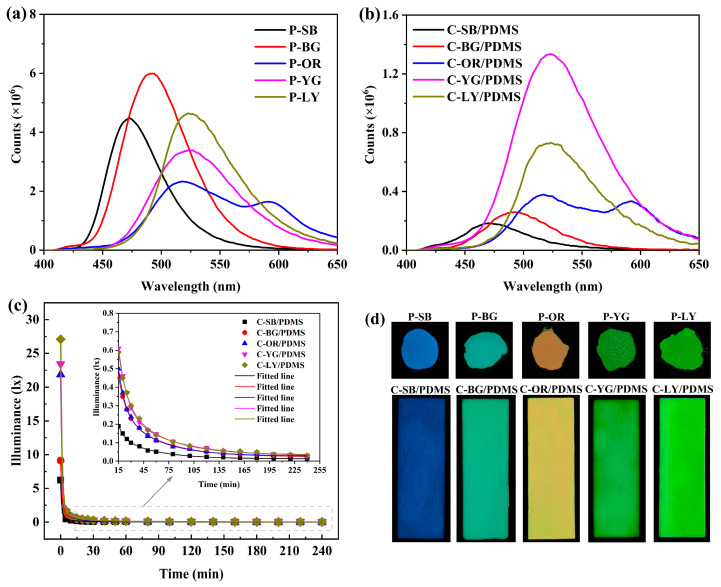
The fluorescence emission spectra (PL) of (**a**) WLAP and (**b**) WLAP/PDMS composite coatings; (**c**) The decay of the afterglow illuminance of coatings; (**d**) The afterglow photos of WLAP and coatings.

The antifouling performance of WLAP/PDMS composite coatings is significantly influenced by the luminous performance of coatings. The PL of WLAP and WLAP/PDMS composite coatings was tested using an excitation wavelength of 370 nm, as presented in Figure 5a,b, respectively. It can be observed from Figure 5a that P-SB had an emission wavelength of 473 nm; P-BG had a wavelength of 492 nm; and P-OR, P-YG, and P-LY had wavelengths of 516 nm. The fluorescence intensity of WLAP with various colors was different. Even though P-OR, P-YG, and P-LY had the same wavelength, their fluorescence intensity varied noticeably, among which P-LY had the highest intensity, and P-OR had the lowest. P-SB had the highest fluorescence intensity among all powders. In comparison with the PL of WLAP, it can be seen from Figure 5b that the fluorescence intensity of the corresponding coatings increased visibly. This was because the film-forming substance, pigments, and fillers blocked some of the light of WLAP, especially the blue light with a shorter wavelength. Figure 5c illustrates the decay rate of the afterglow illumination of the composite coatings examined by a weak light photometer. It can be seen that the illumination of the coatings decreased exponentially. The coating’s illumination drastically decreased over time from 0–15 min, and the decay rate was gradual from 15–60 min. After 60 min, the illumination decayed gently. The initial illumination and fluorescence intensity of the coatings revealed the same trend. The afterglow photos of WLAP and the coatings are given in Figure 5d.

### 3.6. Antibacterial Performance

**Figure 6:**
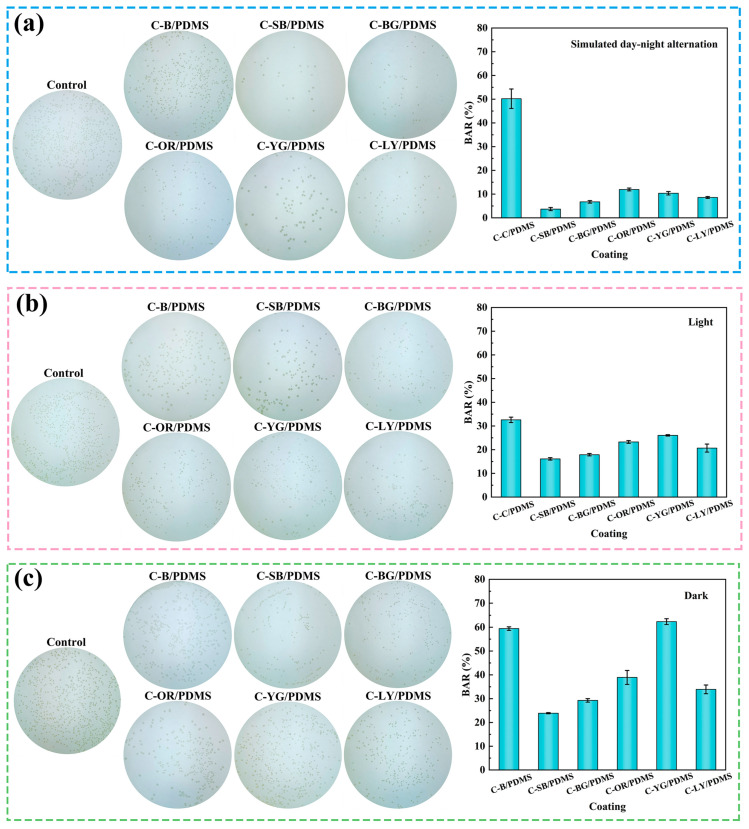
The colony medium image and bacterial adhesion rate (BAR) of coatings under different light conditions: (**a**) Simulated day-night alternation, (**b**) continuous light, and (**c**) continuous dark.

In the ocean, various bacteria can easily attach to the surface of marine facilities. These bacteria produce EPS during the attachment process, forming bacterial mucosa formed [37]. The physicochemical conditions in the mucosa, such as salinity, temperature, dissolved oxygen, and pH value, are quite different from those in the surrounding environment, providing good conditions for the reproduction and growth of microorganisms, encouraging marine algae, spores, larvae, and other organisms to further grow, develop, and reproduce on the mucosal surface, and promoting the colonization of large biofouling organisms such as oysters, mussels, and barnacles [38]. Hence, evaluating the antibacterial effects of coatings is very essential. The antibacterial properties of coatings were tested under simulated day-night alternation, constant light, and continuous dark conditions, respectively. The colony plate images and the calculation of BAR are presented in Figure 6. It is obvious that the coatings exhibit excellent antibacterial properties under simulated day-night alternation conditions (Figure 6a). C-SB/PDMS had the best antibacterial performance, with a BAR of only 3.7%. The BAR of WLAP/PDMS composite coatings was significantly lower than that of C-B/PDMS, suggesting that the fluorescent effect had obvious antibacterial performance. The weak light emitted by composite coatings can provide energy for photosynthetic microorganisms, making them prioritize photosynthesis and interfering with their normal adhesion behavior. In comparison with other colors, blue light with a 473 nm wavelength has more effective antibacterial effects [39,40]. As a result, the antibacterial performance of C-SB/PDMS was the best and could effectually inhibit the initial adhesion of marine bacteria.

It can be seen from Figure 6b,c that the BAR of WLAP/PDMS composite coatings under continuous light or dark conditions significantly increased compared with that under simulated day-night alternation conditions, indicating that the weak light emitted by the coatings can inhibit the attachment of bacteria more efficiently. Moreover, it can be noticed that the BAR under light conditions had relatively reduced compared with that under dark conditions, which indicates that continuous light can affect the attachment of bacteria and further demonstrates that photosynthetic bacteria will perform photosynthesis in the presence of light, hence decreasing adhesion.

### 3.7. Analysis of Antibacterial Mechanism

#### 3.7.1. Simulated Day-Night Alternation

Photosynthetic bacteria, with colors of pink, purple, orange, brown, green, etc., exist in marine bacteria. These bacteria are anaerobic photosynthetic bacteria, which mostly live in anaerobic waters containing hydrogen sulfide and use light energy to reduce CO_2_ into cellular materials [41]. Some bacteria in Rhodospirillum belong to this nutrient type. Therefore, the light emitted by coatings at night can affect the normal physiological behavior of these bacteria, making it difficult for them to attach to marine installations. In addition, light can interact with porphyrins to produce reactive oxygen species (ROS), which can damage the structure of the bacteria and lead to bacterial inactivation [42,43].

**Figure 7:**
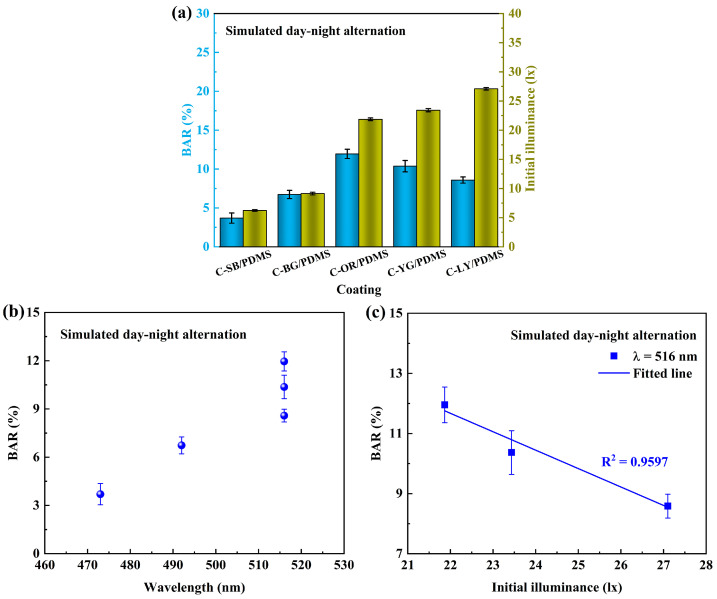
The relationship between BAR under simulated day-night alternation conditions and (**a**) initial illuminance of coatings, (**b**) wavelength of coatings, and (**c**) initial illuminance of C-OR/PDMS, C-YG/PDMS, and C-LY/PDMS with the same wavelength.

Visible light of various wavelengths has been proven to have antibacterial properties [44,45]. Figure 7a demonstrates the relationship between BAR and the initial illuminance of composite coatings under simulated day-night alternation. It can be noticed that C-SB/PDMS with the lowest illuminance showed the best antibacterial performance, suggesting that blue light with a 473 nm wavelength has a more significant antibacterial effect than light of other wavelengths. Relevant studies have shown that light in the blue spectrum region can effectively play a bactericidal role [46]. Blue light at 450–470 nm can induce the production of more ROS, more effectively causing bacterial inactivation [44]. The relationship between BAR and the wavelengths of coatings can be clearly seen in Figure 7b, and it is obvious that the antibacterial performance of shorter wavelengths is better. For coatings with the same wavelength (C-OR/PDMS, C-YG/PDMS, and C-LY/PDMS), their BAR decreased as the initial illuminance increased, which suggests that the high illuminance intensity of coatings is conducive to inhibiting the adhesion of bacteria. Light intensity relates to the total number of photons received per unit area in a given period, while photosynthesis and the production of ROS are fueled by the total number of photons received [42]. Hence, the higher fluorescence intensity at the same wavelength of coatings showed better antibacterial properties.

#### 3.7.2. Continuous Light or Darkness

**Figure 8:**
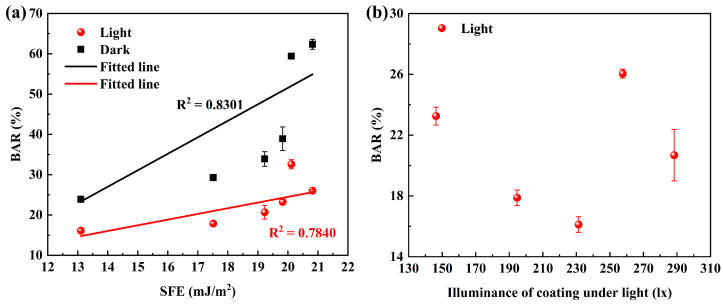
The relationship between (**a**) BAR under continuous light or dark conditions and SFE of coatings (The SFE of coatings shows an increase as follows: C-SB/PDMS, C-BG/PDMS, C-LY/PDMS, C-OR/PDMS, C-B/PDMS, and C-YG/PDMS) and (**b**) BAR and illuminance of coatings under constant light conditions (The illuminance of coatings shows an increase as follows: C-OR/PDMS, C-BG/PDMS, C-SB/PDMS, C-YG/PDMS, and C-LY/PDMS).

Figure 8a shows the relationship between the BAR and SFE of coatings under continuous light or dark conditions. It can be observed that the BAR and SFE of coatings were linearly correlated under light conditions, which proves that the antibacterial performance of coatings is related to the surface properties under this condition. To verify whether the illumination of composite coatings will affect the antibacterial performance under this condition, the illumination of the coating under strong light was tested. The relationship between it and BAR is shown in Figure 7b, and it is evident that there is no linear relationship between them, indicating that the illumination of the coatings was covered under strong light and did not affect the adhesion of bacteria. The main influence factor was the constant strong light source. Under continuous dark conditions (Figure 7a), there was a linear relationship between the BAR and SFE of the coatings, indicating that the antibacterial performance of the coatings is mainly dependent on their surface properties in the absence of fluorescence effects.

## 4. Conclusions

In conclusion, novel WLAP/PDMS composite coatings with the synergetic antifouling effect of fouling-release and fluorescence were developed, significantly enhancing the static antifouling ability of coatings. The antibacterial adhesion experiment demonstrated that under simulated day-night alternation conditions, the light emitted by coatings at night can inhibit the initial adhesion of marine bacteria and exhibit excellent antibacterial properties to avoid subsequent adhesion of fouling organisms. Both light intensity and wavelengths affect the normal activities of marine bacteria. Blue light at 473 nm had the best antibacterial effect, and as a result, the BAR of C-SB/PDMS was only 3.7%. At the same wavelength, high-intensity light can more effectively inhibit bacterial adhesion. The constant light source also influenced the normal adhesion behavior of bacteria and covered the weak light of coatings. In the condition of continuous darkness, the coatings did not have fluorescence effects, and the fouling-release performance of the coatings determined their antibacterial abilities. The waterborne composite coatings designed in this study provide a new strategy for a new generation of eco-friendly and long-acting antifouling coatings.

## Data Availability

The data in this study are contained within the article.

## Supplementary Materials

The following supporting information can be downloaded at: https://www.mdpi.com/article/10.3390/polym15193873/s1, Figure S1: Particle average and size distribution; Figure S2: EDS mapping; Table S1: Polarity force, dispersion force, surface tension, and polarity of water and diiodomethane; Table S2: Volumetric average particle size and areal average particle size; Table S3: Water contact angle (WCA), diiodomethane contact angle (DCA), polarity force, dispersion force, and surface free energy (SFE) of coatings.

## Abbreviation

BAR	Bacterial adhesion rate
CLSM	Confocal laser scanning microscope
C-B/PDMS	PDMS control coating with no WLAP
C-BG/PDMS	Blue-green WLAP/PDMS composite coating
C-LY/PDMS	Lemon-yellow WLAP/PDMS composite coating
C-OR/PDMS	Orange-red WLAP/PDMS composite coating
C-SB/PDMS	Sky-blue WLAP/PDMS composite coating
C-YG/PDMS	Yellow-green WLAP/PDMS composite coating
DBTDL	Dibutyltin dilaurate
EPS	Extracellular polymeric substances
HT-SOE	Hydroxy-terminated silicone oil emulsion
FWHM	Full width at half maxima
PDMS	Polydimethylsiloxane
PL	Fluorescence emission spectra
PTFE	Polytetrafluoroethylene
P-BG	Blue-green WLAP
P-LY	Lemon-yellow WLAP
P-OR	Orange-red WLAP
P-SB	Sky-blue WLAP
P-YG	Yellow-green WLAP
REAP	Rare earth aluminate phosphor
ROS	Reactive oxygen species
SEM	Scanning electron microscope
SLIPS	Slippery liquid-infused porous surface
SSA	Specific surface area
TEOS	Tetraethyl orthosilicate
VOCs	Volatile organic compounds
WCA	Water contact angle
WLAP	Waterproof long afterglow phosphor
XRD	X-ray diffractometer
