# Supplementary material for: Influence of Light Conditions on the Antibacterial Performance and Mechanism of Waterborne Fluorescent Coatings Based on Waterproof Long Afterglow Phosphors/PDMS Composites
Source: Polymers (Basel). 2023 Sep 24;15(19):3873. doi: 10.3390/polym15193873 (PMC10574996; doi:10.3390/polym15193873)
Supplement: Supplementary file 1 [file polymers-15-03873-s001.zip › polymers-2584123-supplementary.pdf]

polymers

MDPI

Supplementary Materials: Influence of light conditions on the antibacterial performance and mechanism of waterborne fluorescent coatings based on waterproof long afterglow phosphors/PDMS composites

Sinan Hao, Yuhong Qi, Zhanping Zhang

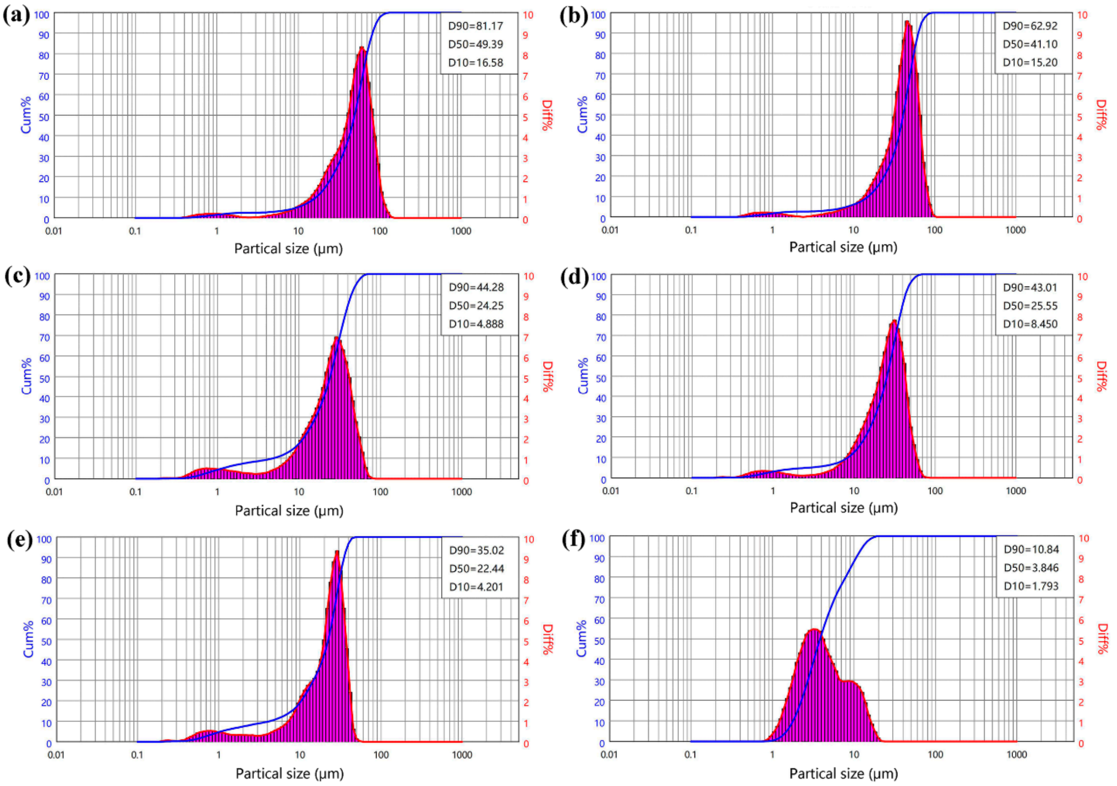

Figure S1. Particle average and size distribution of (a) P-SB, (b) P-BG, (c) P-OR, (d) P-YG, (e) P-LY, and (f) HT-SOE.

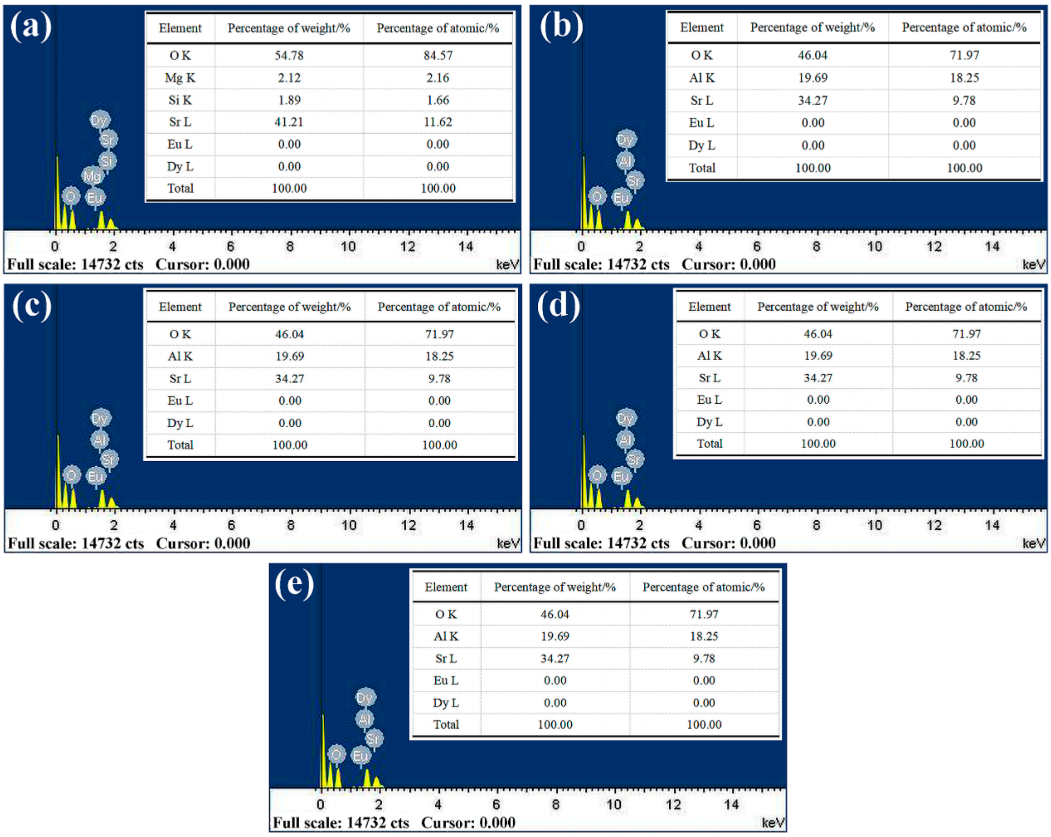

Figure S2. EDS mapping of (a) P-SB, (b) P-BG, (c) P-OR, (d) P-YG, and (e) P-LY.

Table S1. Polarity force γ_L^p, dispersion force γ_L^d, surface tension γ_L, and polarity of water and diiodomethane.

**Liquid**	γ_L^p **/ mJ·m^{-1}**	γ_L^d **/ mJ·m^{-1}**	γ_L **/ mJ·m^{-1}**	**Polarity**
Water	51	21.8	72.8	Polarity
Diiodomethane	2.3	48.5	50.8	Nonpolar

Table S2. Volumetric average particle size and areal average particle size of WLAP and HT-SOE.

**Sample**	**Volumetric average particle size (D[4,3]) /μm**	**Areal average particle size (D[3,2]) /μm**
P-SB	48.66 ± 0.65	17.15 ± 0.12
P-BG	38.85 ± 0.62	14.26 ± 0.50
P-OR	24.80 ± 0.17	6.68 ± 0.06
P-YG	24.11 ± 0.81	8.56 ± 0.19
P-LY	21.48 ± 0.49	6.39 ± 0.18
HT-SOE	5.24 ± 0.08	3.50 ± 0.16

Table S3. Water contact angle (WCA), diiodomethane contact angle (DCA), polarity force γ_s^p, dispersion force γ_s^d, and surface free energy (SFE) of coatings.

**Sample**	**WCA / °**	**DCA / °**	γ_s^p **/ mJ·m^{-2}**	γ_s^d **/ mJ·m^{-2}**	**SFE / mJ·m^{-2}**
C-B/PDMS	100.1 ± 0.86	76.13 ± 2.58	2.40 ± 0.65	17.71 ± 1.62	20.11 ± 1.03
C-SB/PDMS	118.2 ± 1.67	75.55 ± 2.26	0.15 ± 0.04	12.95 ± 1.99	13.10 ± 1.96
C-BG/PDMS	110.4 ± 1.09	81.35 ± 1.11	2.82 ± 0.26	14.69 ± 1.07	17.51 ± 0.61
C-OR/PDMS	99.2 ± 0.71	76.35 ± 0.98	2.44 ± 0.17	17.39 ± 1.48	19.83 ± 0.52
C-YG/PDMS	104.1 ± 1.44	73.85 ± 2.79	0.91 ± 0.09	19.91 ± 3.42	20.83 ± 1.54
C-LY/PDMS	102.0 ± 1.51	77.05 ± 1.44	1.74 ± 0.22	17.49 ± 0.62	19.22 ± 0.80
